# *“I just don’t want to take up people’s time”* - A qualitative study exploring psychological safety during patient–healthcare provider conversations about medications

**DOI:** 10.1371/journal.pone.0356476

**Published:** 2026-08-20

**Authors:** Rasha El-Kotob, Lauren Cadel, Jacob Crawshaw, Saniyah Farzeen, Aleena Ahmad, Lisa Dolovich, Lisa M. McCarthy, Sander L. Hitzig, Diana Zidarov, Crystal MacKay, Stephanie R. Cimino, James Milligan, Aisha Lofters, Sara J.T. Guilcher

**Affiliations:** 1 Leslie Dan Faculty of Pharmacy, University of Toronto, Toronto, ON, Canada; 2 Institute for Better Health, Trillium Health Partners, Mississauga, ON, Canada; 3 Department of Family and Community Medicine, Temerty Faculty of Medicine, University of Toronto, Toronto, ON, Canada; 4 St. John’s Rehab Research Program, Sunnybrook Research Institute, Sunnybrook Health Sciences Centre, North York, ON, Canada; 5 Rehabilitation Sciences Institute, Temerty Faculty of Medicine, University of Toronto, Toronto, Canada; 6 Department of Occupational Science and Occupational Therapy, Temerty Faculty of Medicine, University of Toronto, Toronto, ON, Canada; 7 Dalla Lana School of Public Health, University of Toronto, Toronto, ON, Canada; 8 Institut universitaire sur la réadaptation en déficience physique de Montréal, Centre intégré universitaire de santé et de services sociaux du Centre-Sud-de-l’Île-de-Montréal, Montreal, QC, Canada; 9 Centre de Recherche Interdisciplinaire en Réadaptation du Montréal Métropolitain, QC, Canada; 10 School of Rehabilitation, Faculty of Medicine, Université de Montréal, Montreal, QC, Canada; 11 KITE, Toronto Rehab-University Health Network, Toronto, ON, Canada; 12 Department of Physical Therapy, Temerty Faculty of Medicine, University of Toronto, Toronto, Canada; 13 Faculty of Health Sciences, School of Rehabilitation Therapy, Queen’s University, Kingston, ON, Canada; 14 Lawson Research Institute, St. Joseph’s Healthcare, London, ON, Canada; 15 Faculty of Health Sciences, Department of Family Medicine, McMaster University, Hamilton, ON, Canada; 16 Peter Gilgan Centre for Women’s Cancers, Women’s College Hospital, Toronto, ON, Canada; UTM Skudai: Universiti Teknologi Malaysia, MALAYSIA

## Abstract

**Background:**

Psychologically safe environments, in which patients feel respected, able to ask questions, and comfortable raising concerns, are key aspects of person-centered care and overall quality of care. Examining the psychological aspect of managing medications remains underexplored despite its potential to improve patient safety and quality of care. To date, there is limited research on the perceptions of psychological safety among patients when discussing medications with their healthcare providers (HCPs). To address this gap, we explored perceptions of patient psychological safety during clinical encounters for medication management within primary and community care settings.

**Methods:**

We conducted a qualitative study in Ontario, Canada, with adults taking at least one medication for ≥3 months. Participants were recruited through purposive, convenience, and snowball sampling. Semi-structured interviews and focus groups were held virtually between May and August 2024. The Patient Psychological Safety framework, which includes the constructs of ‘Patient Belonging’ (feeling accepted), ‘Patient Learning’ (feeling encouraged to ask questions), and ‘Patient Participating’ (feeling safe to share opinions), was used to guide a secondary deductive content analysis.

**Results:**

Twenty-one individuals participated; most were ≥40 years old (n = 19), women (n = 14), and White (n = 14). Within ‘Patient Belonging’, participants described that feeling heard by HCPs supported perceptions of safety, whereas being dismissed contributed to mistrust. For ‘Patient Learning’, participants indicated that clear and contextualized medication communication reduced anxiety and distress; in contrast, feeling overwhelmed, embarrassed or anxious during healthcare encounters acted as barriers to learning. For ‘Patient Participating’, participants reported feeling empowered when invited to engage in medication-related decision-making, while perceived exclusion by HCPs led to frustration and disengagement.

**Conclusion:**

Psychological safety is integral to effective conversations about medications between patients and HCPs. Fostering environments in which patients feel heard, respected, and able to contribute to clinical decision-making may reduce distress and support engagement in medication treatment decisions. Future work should examine strategies to address multi-level barriers to promoting psychologically safe healthcare encounters around medications.

## 1. Introduction

In light of the widespread global use of pharmacotherapy [1], the World Health Organization (WHO) has identified person-centered medication management and medication safety as priorities [2,3]. Psychological safety is a key component of person-centered care and reflects a positive therapeutic alliance between patients and healthcare providers (HCPs) [4,5]. Psychological safety involves fostering an environment where people feel confident voicing their health concerns and expressing their opinions without fear of negative repercussions [5,6]. In the context of medication management, fostering psychological safety may enhance communication about medications and encourage patients to speak up about concerns or errors related to their medications [7].

Medication management can be complex, involving interactions with multiple individuals including patients, care partners, prescribers, pharmacists, and other HCPs, which may increase the likelihood of medication errors [8–10]. These interactions are particularly complex in primary and community care settings, where medication-related decisions are often made and revisited over time and are distributed across multiple providers and settings. Consequentially, primary and community care settings are critical contexts for advancing patient psychological safety in medication management, as most medication-related decisions, monitoring, and everyday use occur in these settings [11]. Unlike in hospital settings, care in primary and community settings is often fragmented, with patients serving as the most consistent presence across care encounters [12]. Patients are also primarily responsible for self-administering and managing medications, which increases the need for psychological safety [13].

In primary and community care, patient–provider interactions are often longitudinal and relationship-based [6,14–16]. The attitudes and communication styles adopted by HCPs may affect the emotions patients experience and their ability to voice concerns regarding their medications, potentially impacting medication adherence [6]. Previous research has shown that if HCPs adopt a perceived judgmental or dismissive attitude, it may prompt negative emotions among patients and hinder honest conversations, potentially leading to unsafe medication use [14]. Conversely, compassionate communication can promote perceptions of psychological safety required for discussing effective medication use [6].

Importantly, the psychological aspect of medication management remains an important but underdeveloped topic [3,17]. The Patient Psychological Safety (PPS) framework has been developed to characterize how patients experience psychological safety in healthcare, including dimensions related to belonging, learning, and participation [4]. However, the application of such frameworks to medication-related encounters remains limited, particularly from the patient perspective. A better understanding of patients’ experiences of psychological safety in medication-related encounters may support diagnostic and treatment plans that align with patients’ needs and preferences, improving the overall quality of care and patient safety [18]. Therefore, using the PPS framework, our study objective was to explore perceptions of patient psychological safety during clinical encounters for medication management within primary and community care settings.

## 2. Materials and methods

### 2.1. Study design and research paradigm

This study was situated within a larger three-phase, mixed-methods research project exploring perspectives and implementation considerations of a patient-reported experience measure for medications (PREM-Rx) among patients (persons with lived experience of taking medications), HCPs, and decision-makers (administrators involved in medication-related policy decisions) [19]. Using a post-positivist approach [20], we conducted a qualitative analysis to explore patients’ experiences of psychological safety in patient-HCP interactions related to medications. The reporting of this study aligns with the Standards for Reporting Qualitative Research Checklist (see S1 Table) [21].

### 2.2. Patient psychological safety framework

We used the PPS framework proposed by Rathert and colleagues [4] to guide our secondary analysis of the PREM-Rx dataset. This framework is informed by prior literature [22–24]. The PPS framework includes three key domains: 1) ‘Patient Belonging’, which captures how patients feel accepted, respected, and welcomed without being dismissed, 2) ‘Patient Learning’, where patients are encouraged to ask questions, admit mistakes, and receive clear and understandable answers from their HCPs, and 3) ‘Patient Participating’, which emphasizes the importance of patients feeling safe to share their opinions on their diagnosis or treatment, having a say in their care, and being invited to actively engage in decision-making [22–24]. This framework provided a structured and deductive approach to examine patients’ experiences of psychological safety in medication-related interactions.

### 2.3. Context

This study was conducted in Ontario, Canada, which has a population of approximately 16 million people [25]. Approximately 20% of residents have public prescription medication coverage, ~55% rely on employer-sponsored plans, and ~5% use private coverage [26,27]. Ontario provides a relevant setting to explore psychological safety in medication-related encounters within a publicly funded healthcare system (for medically necessary physician and hospital services) alongside mixed public-private drug coverage [19].

### 2.4. Study participants and recruitment

Participants were adults living in Ontario who had taken at least one medication prescribed by a regulated HCP for a minimum of three months. Recruitment used purposive, convenience, and snowball sampling, including outreach to a participant pool from previous projects [19]. We aimed to achieve variation across demographic characteristics (e.g., age, sex, gender, role, education). Participants were recruited online and in-person through social media, community organizations, and organizational listservs, websites, and newsletters [19].

### 2.5. Researcher characteristics and reflexivity

The research team included members at multiple career stages with expertise in pharmacy, qualitative health research, rehabilitation sciences, family medicine, and implementation science. The interviewers (RE and LC) were trained qualitative researchers with experience conducting semi-structured interviews and focus groups. Throughout data collection and analysis, the multidisciplinary research team met regularly to discuss emerging interpretations and reflect on how their disciplinary perspectives and prior experience in medication-related research may have influenced interpretation of the findings.

### 2.6. Data collection

Qualitative data were collected through semi-structured interviews and focus groups [19]. The interview guide was informed by domains from the Theoretical Domains Framework (TDF) to explore experiences with medications and implementation considerations for reporting medication-related experiences [19,28,29]. Interviews and focus groups were conducted between 01/05/2024–31/08/2024 by two trained qualitative researchers (RE and LC) [19]. Sessions were conducted via Zoom or telephone, lasted up to 60 minutes, and were audio-recorded and transcribed [19]. Participants received an honorarium for participation. Data collection continued until information power was considered sufficient [30]. Throughout data collection, the research team regularly assessed whether the interview data were sufficiently rich and relevant to address the study objectives, and decisions regarding sample adequacy were made collaboratively through ongoing discussions. As this study represents a secondary analysis of an existing qualitative dataset, we additionally considered whether the available interview data contained sufficient depth and breadth to address the present research question.

### 2.7. Data analysis

In the larger mixed-methods study described above, a rapid qualitative analysis was conducted following a similar approach to Nevedal and colleagues [31]. Interview data were initially deductively coded using a structured matrix aligned with domains from the TDF, with additional inductive codes added as needed. During this analysis, concepts related to patient psychological safety  consistently emerged across participant interviews but were not explored in depth because the primary analysis focused on barriers and facilitators to PREM-Rx implementation. Given the prominence of these concepts across participant groups, we conducted a secondary deductive analysis of the existing qualitative dataset using the PPS framework. A data display matrix, aligned with PPS constructs (patient belonging, learning, and participating; see S2 Table), was developed in Microsoft Excel. The primary analyst (SF or AA) coded transcripts into the matrix, and a secondary analyst (RE or AA) reviewed the coded transcripts, supplemented the coding where appropriate, and discussed any differences in interpretation with the primary analyst until consensus was achieved. Analysts met weekly to discuss coding decisions and refine the coding framework throughout the analysis.

### 2.8. Ethical considerations and reporting

The study received ethics approval from the University of Toronto Research Ethics Board (#46159). All participants provided written informed consent prior to participation.

## 3. Results

A total of 21 participants were included. The majority were aged 40 years or older (n = 19), White (n = 14), female (n = 16), and identified as women (n = 14) (see Table 1). Most data were collected via one-on-one interviews, rather than focus groups, due to the sensitive nature of the topic. Findings are presented across three constructs of the PPS framework: patient belonging, learning, and participating.

**Table 1:** Participant characteristics with lived experience taking medications (n = 21).

Characteristic	Participants, n, (%)
**Age**	
18-39	2 (9.5)
40-59	11 (52.4)
60+	8 (38.1)
**Gender**	
Woman	14 (66.7)
Man	5 (23.8)
Non-binary or transgender	2 (9.5)
**Sex**	
Female	16 (76.2)
Male	5 (23.8)
**Race**	
Black	2 (9.5)
Indigenous	1 (4.8)
South Asian	1 (4.8)
Southeast Asian	1 (4.8)
White	14 (66.7)
Unspecified	2 (9.5)

### 3.1. Patient belonging: Acceptance and respect during healthcare encounters

Participants described varied experiences of belonging during medication-related healthcare encounters, including feeling accepted, respected, and heard. When present, these experiences foster trust and emotional comfort, particularly during times of distress. One participant explained: *“I try to treat providers respectfully and behave in an intelligent way with them too. And they treat me as an equal”* [P10]. Another participant noted: *“My doctor is very good at asking me about how I am, my experiences, my concerns, and [their] reason for prescribing things [medications] or changing things [medications]”* [P09].

Conversely, some participants described feeling dismissed or disrespected by HCPs. One participant expressed: “*Okay, a big thing that has been present in this last few years is the health care system trying to solve the opiate crisis on the backs of pain patients, and so I was pulled off my pain medications after 10 years of successful pain management. And my life went to [expletive], I was disbelieved and dismissed”* [P02]. Several participants discussed perceptions of being dismissed that seemed to be further compounded by their social location such as race, gender, and disability. One participant expressed how her racial identity influenced how HCPs perceived her: “*I’m a Black woman and right now, they’re only seeing me in this disease state... Assumptions are being made based on how I’m physically presenting to them*” [P03]. Similar frustrations were described by a participant with autism: *“Ableism. Yep, I get that all the time. ‘You’re high functioning so you can do this thing [fill out a medical form].’ I’m like, ‘No, I can’t do that thing [fill out a medical form]. Just because I’m functioning doesn’t mean I can do that thing [fill out a medical form].’ Yeah, forms are hard. Forms are very hard. I have to fill out a form to get access to a doctor or a medication or a specialist, like it’s ridiculous*” [P06]. These accounts highlight how experiences of dismissal were often shaped by intersecting social identities. Overall, psychological safety was closely tied to patients feeling respected, heard, and that care was tailored to their needs.

### 3.2. Patient learning: Understanding and education

Participants described diverse learning needs and preferences when engaging with medication-related information. Some participants felt empowered by proactively researching information about their medications, while others described self-educating as overwhelming and expressed a need for additional support from HCPs. One participant explained: *“I like to research things [medication information] on the internet on my own and then I’ll send him [HCP] articles... and then he’ll take a look”* [P07]. In contrast, another participant noted: *“I don’t mind picking up a paper and reading it, but I don’t know nearly enough about the context to understand [medication information]”* [P12].

Participants expressed a strong desire for more information about their medications, which was not always met by HCPs. A participant described: *“I want to know how they’re using that [medication] information. What is what I’ve shared with you mean to the decisions you’re going to make or the guidance directions you’re going to give me in terms of how to manage, right?”* [P03]. Another participant shared: *“I think the one concern I always have is, ‘What are the contraindications of various medications’ and they [HCPs] never tell you that, you know... I just wish there was a little more of an answer, you know, just to satisfy my curiosity... I think I get the feeling sometimes that it’s ‘Oh, you don’t need to know that’ you know, from the medical people”* [P11].

Participants also described embarrassment and doubt when seeking medication information. A participant explained: *“At least in my experience, I felt like embarrassed to ask if, like a medication could cause weight gain or something. It seems like a shallow thing to care about when you’re looking for medication for something, but it’s just part of life in society that makes us think about these things”* [P15]. Stigma surrounding certain conditions or medications (e.g., chronic pain medication, cannabis use) contributed to feelings of shame and hesitation among patients when communicating with HCPs. A participant described: *“Embarrassment. Shame… like it’s from the stigma of having [to take] certain kind of things [medications]. Hesitation. Shyness. And I don’t, I don’t know the emotion, but it’s just like a hesitation”* [P18].

Overall, participants wanted to understand their medications but often felt disempowered when information was unclear, withheld, or difficult to interpret. Barriers to learning included feeling overwhelmed, embarrassed, or uncertain when discussing medication-related information during healthcare encounters.

### 3.3. Patient participating: Involvement in decision-making

Most participants valued involvement in medication-related decision-making and wanted to be actively engaged in their care decisions. For example, one participant explained: *“I want to be a part of the decisions that are made about my care and my treatment including the medications I’m prescribed. So that I can better learn the what and the why and what to look out for”* [P03]. Another participant noted: *“Because of Western medicine’s reliance on medication as a form of healthcare, if it’s going to be the primary thing, I better have some input on it”* [P18].

However, some participants were frustrated by being excluded from decision-making. One participant shared: *“I feel like they have this entitlement to tell me what will work or what won’t work when I’m the only one who can say it did work, or it didn’t work [the medication]”* [P01]. Some participants felt comfortable advocating for their medication treatment: *“I would talk to my doctor and say, ‘I don’t believe this medication is working, so I want the bloodwork to make sure the levels are right.’ So I have no problem confronting them and telling how I feel... I would say ‘I feel I should be on this medication and these are the reasons why’”* [P20]. For others, having a patient advocate present during consultations might increase confidence in engaging in discussions: *“Doctors tend to act a little bit differently when someone else is in the room... it would have like given me confidence [to discuss medication treatment plans]”* [P02].

Feelings of uncertainty or emotional distress discouraged participants from engaging in decision-making with their HCPs. One participant described: *“Before I had that [negative] experience, I would say, knowing that my doctor was gonna hear me and try to help made it so that I never kept anything from [them] which is to me just what patient and doctor relationships should look like. Now, I second guess myself, or… it’s hard to raise your voice if you have this anticipation”* [P02]. There was also uncertainty regarding who patients should talk to when having issues with their medications and whether their concerns are valid. One participant noted: *“I think I would want to be clear as to who I would talk to, or which body I would go to when having issues [with medications] because right now I don’t really know”* [P10]*.* Another participant questioned: *“Am I going to sound stupid reporting something [about my medications]? Is this like, everybody else knows this?”* [P08].

Conversely, when participants were encouraged to be involved in decision-making, they described a stronger sense of partnership with their HCPs, which facilitated open communication. For instance, one participant described how their HCP invited input: *“‘What do you think [about medication use]? Here are the benefits, here are the drawbacks, how are you going to proceed?’”* [P14]. Another participant offered: *“I have a great nurse practitioner and pharmacist. So anytime I have an issue, I can call either of them and talk to them about what I’m experiencing [with medications]. And my pharmacist even goes as far as to call me and say, ‘Look, this is the medication that so-and-so wants to put you on, but it’s going to interact with this, this, and this [medication], so we’re going to fax them and tell them this is what you need.’”* [P04].

Overall, participation in decision-making ranged from exclusion to active partnership and was shaped by both HCP engagement and patients’ confidence in raising concerns. Active involvement in decision-making was more commonly described when participants felt supported in expressing concerns and asking questions.

## 4. Discussion

In the present study, psychological safety in medication-related encounters was shaped by three interrelated constructs outlined in the PPS framework: trust in HCPs, feeling well-informed about medications, and active involvement in decision-making. These constructs appear to operate together, supporting psychologically safe interactions that may enable safer and more effective medication management. Medication-related encounters may be particularly vulnerable to breakdowns in psychological safety in primary and community care, as they are often brief, information-dense, and occur across fragmented care settings involving multiple providers [12,32]. This context helps to explain why trust, communication, and patient involvement emerged as key features that patients value in medication-related encounters.

A key finding was that respectful and empathetic interactions with HCPs foster a sense of belonging. These findings align with prior research showing that inclusive, empathetic communication supports patient trust, engagement, and improved health outcomes [33,34]. In our study, several participants described feeling dismissed or disrespected by HCPs, reinforcing the importance of communication that is inclusive and responsive to patients’ needs in fostering a sense of belonging and psychological safety [35].

Another key finding was the importance of effective communication in supporting patient learning and engagement. In our study, participants described how unclear or incomplete communication about medications contributed to feelings of disempowerment and embarrassment which limited their willingness to ask questions or seek clarification. This aligns with prior work demonstrating that accessible, empathetic communication supports patient understanding and engagement when managing medications [36–38].

Psychological safety within healthcare settings may also support agency and self-management [7]. Our findings showed that patients want to be involved in medication-related decision-making but some described feeling discouraged during healthcare encounters. In our study, participants who were actively included in decision-making described stronger partnerships with their HCPs, which facilitated open discussions about medications. Prior research similarly suggests that psychologically safe environments enable patients to express concerns and engage more confidently in their care [7,39,40]. However, building trust through collaborative relationships requires time, which may be limited in primary and community care settings and under current remuneration models [41,42]. These structural constraints may limit opportunities to develop psychologically safe environments that support patient participation in medication-related decisions.

From a practical perspective, fostering psychological safety during medication-related encounters may require action at both HCP and healthcare system levels. HCPs can support psychological safety by creating opportunities for patients to ask questions, acknowledging medication-related concerns without judgement, providing clear and tailored information, and actively involving patients in medication-related decision-making. At the systems level, healthcare organizations may benefit from considering how factors such as appointment duration, continuity of care, and communication skills training may influence the ability of HCPs to create psychologically safe medication-related encounters.

### 4.1. Future research directions

Future research should explore the intersection of psychological safety and multi-level factors such as social determinants of health (e.g., race, gender, socioeconomic status, and disability) and the relationship of these factors to medication experiences and outcomes [43,44]. Examining how identity-related factors are compounded by patients’ experiences of psychological safety within healthcare settings may inform targeted interventions that reduce healthcare disparities and improve patient safety and care. Based on our previous research [19] and additional literature [41,45,36], it is important to consider that other factors may prevent patients from learning about their medications during healthcare encounters. These factors are myriad at the micro-level (e.g., language and cultural barriers) [19,46], meso-level (e.g., short medical appointments with insufficient time for adequate patient learning, lack of training and support for HCPs) [19], and macro-level (e.g., insufficient health resources such as translation services for patients and inadequate health financing) [46]. Future work should therefore explore strategies to support psychologically safe healthcare environments while addressing these multi-level barriers [41].

Future research should also further develop the theoretical understanding of patient psychological safety. While the PPS framework provides a useful structure for categorizing patient experiences, it is inherently parsimonious and does not explicitly articulate relationships between constructs or the mechanisms through which psychological safety influences communication, learning, and participation. Future work could build on the PPS framework by examining how its constructs interact over time and by integrating behavioural or implementation science theories to better explain how psychological safety shapes medication-related behaviours, experiences, and outcomes.

### 4.2. Study limitations and strengths

This study had several limitations. Most participants identified as White women aged 40 years or older despite efforts to recruit a diverse sample. Consequently, the findings may not fully reflect the experiences of younger adults, men, or individuals from underrepresented racial and ethnic groups, limiting the transferability of the findings to these populations. Furthermore, recruitment through purposive, convenience, and snowball sampling may have favored individuals who were more engaged with their healthcare and more willing to discuss their medication experiences. Consequently, the perspectives of people who are less engaged with healthcare services or less comfortable participating in research may be underrepresented. Additionally, all participants were recruited from Ontario, Canada, which may limit transferability to other populations and healthcare settings. Despite these limitations, this study also has several strengths. The use of in-depth interviews and focus groups provided rich insights into patients’ experiences discussing medications during healthcare encounters. In addition, applying the PPS framework offered a structured approach to understanding psychological safety within medication-related interactions.

## 5. Conclusion

Patients may be more willing to discuss medication-related experiences when they feel psychologically safe during healthcare encounters. Our findings suggest that respectful patient-provider interactions, clear and empathetic communication, and active involvement in decision-making may support psychologically safe discussions about medications. Creating psychologically safe environments for medication-related conversations may strengthen communication, engagement, and person-centered care within primary and community care settings. Further research is needed to identify strategies that support psychologically safe medication-related encounters in real-world clinical practice.

## Supporting information

S1 Table Standards for Reporting Qualitative Research Checklist. (DOCX)

S2 Table Coding Matrix Example Using the Patient Psychological Safety Framework. (DOCX)
