# Supplementary material for: “I just don’t want to take up people’s time” - A qualitative study exploring psychological safety during patient–healthcare provider conversations about medications
Source: PLoS One. 2026 Aug 20;21(8):e0356476. doi: 10.1371/journal.pone.0356476 (PMC13492807; doi:10.1371/journal.pone.0356476)
Supplement: Supplementary file 2 [file pone.0356476.s002.docx]

**Supplementary Table 2.** Coding Matrix Example Using the Patient Psychological Safety Framework

| **Participant #** | **PPS Construct** | **Summary Point** | **Quotes** |
| --- | --- | --- | --- |
| P03 | Patient Belonging | Participant felt dismissed and disrespected during medical appointments. They felt that HCPs were making assumptions based on their race and health condition. | “That [being dismissed] didn't make me feel good at all. Didn't make me feel seen or respected.”  “I feel like some of them [HCPs] just treat me like a textbook case and I know I'm not.” |
|  | Patient Learning | Participant expressed desire to learn about their medications due to potential benefits. They requested more information about their medications to make informed decisions. | “I want to be a part of the decisions that are made about my care and my treatment including the medications I'm prescribed. So that I can better learn the what and the why and what to look out for.”  “I want to know how they're using that information. What is what I've shared with you mean to the decisions you're going to make or the guidance and directions you're going to give me in terms of how to manage [medications], right?” |
|  | Patient Participating | Participant felt excluded from care decisions by their HCPs. | “I asked them [specialist] specifically, questions... they gave my question in response to the resident, not me.”  “I'm not being involved in or made aware of the decisions that are being made about my care by him [specialist].” |

*P = Patient; HCP = Healthcare provider
